# Rotational Mechanism of F_O_ Motor in the F-Type ATP Synthase Driven by the Proton Motive Force

**DOI:** 10.3389/fmicb.2022.872565

**Published:** 2022-06-16

**Authors:** Shintaroh Kubo, Shoji Takada

**Affiliations:** ^1^Department of Anatomy and Cell Biology, McGill University, Montreal, QC, Canada; ^2^Department of Biophysics, Graduate School of Science, Kyoto University, Kyoto, Japan

**Keywords:** F_O_F_1_ ATP synthases, F_O_ motor, molecular dynamics simulations, coarse-grained model, Monte Carlo simulations

## Abstract

In F_O_F_1_ ATP synthase, driven by the proton motive force across the membrane, the F_O_ motor rotates the central rotor and induces conformational changes in the F_1_ motor, resulting in ATP synthesis. Recently, many near-atomic resolution structural models have been obtained using cryo-electron microscopy. Despite high resolution, however, static information alone cannot elucidate how and where the protons pass through the F_O_ and how proton passage is coupled to F_O_ rotation. Here, we review theoretical and computational studies based on F_O_ structure models. All-atom molecular dynamics (MD) simulations elucidated changes in the protonation/deprotonation of glutamate—the protein-carrier residue—during rotation and revealed the protonation states that form the “water wire” required for long-range proton hopping. Coarse-grained MD simulations unveiled a free energy surface based on the protonation state and rotational angle of the rotor. Hybrid Monte Carlo and MD simulations showed how proton transfer is coupled to rotation.

## Introduction

**Figure 1:**
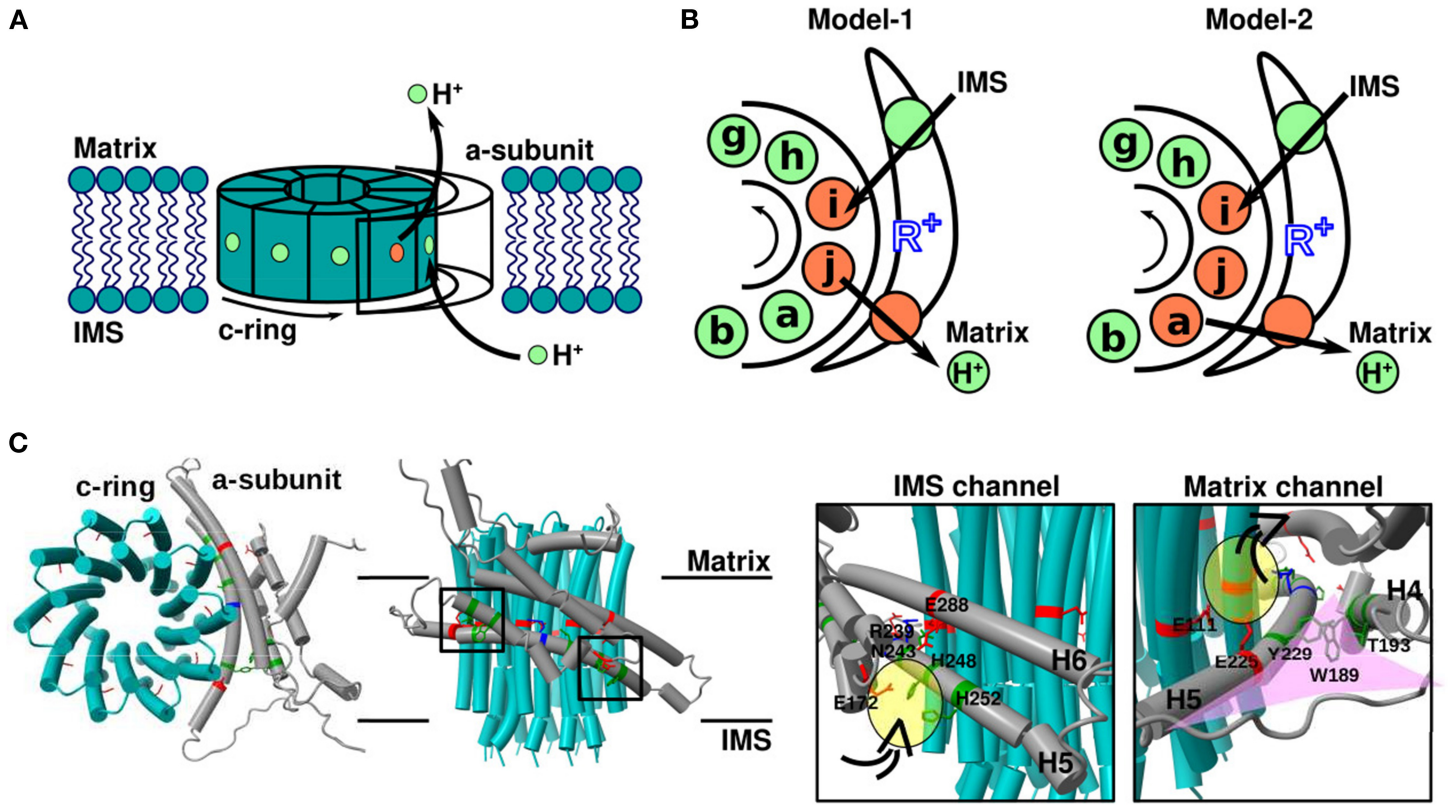
Overview of the F_O_ motor. **(A)** Cartoon showing the geometry of the mitochondrial F_O_ motor. The rotational direction of the c-ring and movement of protons required for ATP synthesis are indicated. **(B)** Cartoons showing the mechanism of proton transfer between the a-subunit and c-ring. Green and orange spheres represent protonated (EH) and deprotonated (E^−^) glutamate, respectively. Black arrows indicate the net proton flow during ATP synthesis. R^+^ (blue) is a highly conserved arginine residue of the a-subunit. **(C)** Top (left) and side (middle) views of the mitochondrial F_O_ motor a-subunit (gray), and c-ring (cyan) (PDB ID: 6F36). Close-up (right) views of the square region in the middle cartoon. Representative residues with acidic, basic, and neutral side chains are indicated in red, blue, and green, respectively. The proton-carrier residue of c-subunit is cE111, the conserved arginine of a-subunit is aR239, and the representative residues of two half-channels are aE288 for the IMS channel and aE225 for the matrix channel. Water-accessible regions in the two half-channels, namely the IMS and matrix channels, are shown in yellow. The funnel-like space in the matrix channel is shown in pink.

F_O_F_1_ ATP synthase, a ubiquitous enzyme that synthesizes most ATP in living cells, comprises two rotary motors: the membrane-embedded proton-driven F_O_ motor and the catalytic F_1_ motor; these motors share the central rotor and peripheral stalk. The F_O_ motor harbors a ring-shaped c-subunit oligomer, which serves as the rotor, and an a-subunit (stator), which mediates proton transfer between the c-ring and outer membrane aqueous environment [matrix and intermembrane space (IMS) of mitochondrial ATP synthase]. The a-subunit possesses two half-channels [confirmed for bacterial, mitochondrial (Srivastava et al., 2018; Spikes et al., 2020), and chloroplast (Hahn et al., 2018) F_O_ motors]. One exchanges protons with IMS or outside the cell, and the other shuttles protons between the matrix and cytoplasm. Since these two half-channels are not directly connected, protons can only pass through the membrane *via* rotation of the c-ring. Therefore, the passage of protons through the membrane is coupled to the rotational motion of the c-ring (Figure 1A).

Recent cryo-electron microscopic (EM) studies have elucidated details of F_O_ rotary mechanisms. For instance, Klusch et al. (2017) confirmed that the two half-channels of the a-subunit harbor continuous spaces through which protons can pass from the matrix/IMS region to the key residues responsible for proton transport in the c-subunit. In the c-ring, the key residues for proton transport receive and pass protons *via* protonation and deprotonation, respectively. Pogoryelov et al. (2010) elucidated the c-ring structure of F_O_ in *Spirulina platensis* and reported that the key residues are in a proton-locked conformation in the lipid membrane environment and in a proton-unlocked conformation in the hydrophilic environment, assuming an interface with the a-subunit. In addition, molecular dynamics (MD) simulations confirmed that these two states could move back and forth depending on the surrounding environment. Thus, high-resolution structures, together with MD simulations, have offered important insights into the proton pathway.

In the present review, we describe theoretical and computational studies aimed at clarifying the rotational mechanisms of the F_O_ motor in F_O_F_1_ ATP synthase. First, we present a chronological overview of the structural and theoretical studies on F_O_. Next, we introduce a recent all-atom MD simulation study that examines how the conformation of side chains of the key residues changes depending on the protonation state and environment. All-atom MD simulations have provided substantial information on water molecule accessibility. The free energy profile of a transferring proton, including its pKa value, is briefly discussed. Then, we describe coarse-grained MD simulations, which allow for modeling large-scale dynamics. Coarse-grained MD simulations have revealed protonation state-dependent free energy surfaces. We explain a computational methodology to simulate proton transfer-coupled F_O_ rotation through a hybrid Monte Carlo/MD approach, which identified a preferred pathway in which two or three c-subunit key residues are deprotonated during a single rotational step. A recent biochemical experiment, together with the corresponding simulations, supports this view. Finally, we discuss the effects of a mutation of the conserved arginine of the a-subunit, the symmetry mismatch between F_O_ and F_1_, and the coupling between side-chain reorientation of the c-subunit key residues and proton transfer-coupled F_O_ rotation.

## Overview of Theoretical Models of the F_O_ Motor

As the fundamental mechanism of proton transfer-driven F_O_ rotation, the model first proposed by Vik and Antonio (1994) has been widely accepted (Figure 1A). The model assumes the presence of two half-channels in F_O_: one that shuttles protons from the IMS aqueous environment to the middle of the membrane, and the other that connects the middle of the membrane to the matrix-side aqueous environment. However, the two half-channels are not directly connected to the membrane. Instead, the end of each half-channel is close to a proton carrier amino acid in the middle of the F_O_ c-subunit such that the proton can be transferred between each half channel and the proton-carrier residue in the c-subunit. Importantly, the c-subunits connected to the two half-channels are not the same in the c-ring. Thus, for the proton to move across the membrane, c-ring rotation is obligatory. Vik and Antonio introduced various mutations in the half-channel residues on the cytoplasmic side of the a-subunit (aE219 in *Escherichia coli*) adjacent to the proton carrier acidic residue of the c-ring (cD61 in *E. coli*) and measured ATP hydrolysis-driven proton pump activity; the authors noted that the proton-accepting residue at the aE219 position is critical for proton transfer. Subsequently, Oster et al. extended the Vik and Antonio model to a quantitative mathematical model (Elston et al., 1998). In this model, the authors focused on switches in electrostatic attractions between protonated and deprotonated acidic residues of the c-ring (cD61 in *E. coli*) and the conserved arginine of the a-subunit (aR210 in *E. coli*), which serves key regulatory roles in enzyme function (Figure 1B, left). This model was also applied to sodium-transporting F_O_F_1_-ATPase, suggesting that it captures the universal nature of the F_O_ motor (Dimroth et al., 1999; Xing et al., 2004). Together, the results successfully explained the experimental data (Vik and Antonio, 1994), and many subsequent theoretical studies were based on these models. For example, Aksimentiev et al. used a low-resolution structure (Girvin et al., 1998; Rastogi and Girvin, 1999) which allowed for speculating the positional association between c-ring and a-subunit for their simulations. Consequently, in addition to distance, the angle of the c-ring relative to the conserved arginine of the a-subunit garnered attention, and their kinetic model was constructed to explain the experimental results (Aksimentiev et al., 2004). In summary, it was theoretically confirmed that the F_O_ motor achieves rotational motion by the kinetics of proton transfer and the associated effects of electrostatic interactions changing.

Then, cryo-EM revolutionized the resolution of structural information. In 2015, cryo-EM structural models of the F_O_ part were obtained at a resolution of ~7 Å (Allegretti et al., 2015; Zhou et al., 2015). Interestingly, the a-subunit of F_O_ harbors two long helices within the membrane, which are highly tilted from the normal to the membrane surface, and the conserved arginine residue is located in the middle of these helices (Figure 1C). This structural model clearly support the half-channel model of Vik and Antonio (1994). Based on the structural information at that time, Warshel group performed coarse-grained MD simulations to evaluate the free energy surface depending on the rotation angle of the c-ring under fixed protonated/deprotonated conditions (Mukherjee and Warshel, 2011). In addition, more recent advances in cryo-EM allowed for obtaining the structure of F_O_ in mitochondrial, chloroplast, and bacterial ATP synthases at a resolution of ~3–4 Å (Guo et al., 2017; Klusch et al., 2017; Hahn et al., 2018; Srivastava et al., 2018; Gu et al., 2019). With the higher resolution models, Warshel group re-performed similar coarse-grained MD simulations to more reliably evaluate the free energy surface (Bai and Warshel, 2019) because their earlier work assumed a different orientation of the a-subunit with respect to the c-ring. Moreover, Roh et al. (2020) and Marciniak et al. (2022) performed all-atom MD simulations to evaluate the free energy surface of F_O_ and V_O_ motors, respectively. We also performed hybrid Metropolis Monte Carlo (MC)/MD simulations of proton transfer-coupled F_O_ rotation, where proton transfer was mimicked by MC moves and the rotational motion of the F_O_ motor was simulated by MD (Kubo et al., 2020). This method is based on the constant-pH MD (Baptista et al., 2002; Mongan et al., 2004) and empirical valence bond (EVB) approaches (Kato et al., 2006; Olsson and Warshel, 2006), and similar approach, as called MCCE approach, used to study conformationally-dependent protonation states in Complex I (Khaniya et al., 2020). Note that as shown in Figure 1B (left), the proton-carrier residue in only one or two c-subunits can be deprotonated under rotation in the conventional model. For instance, Leone and Faraldo-Gómez (2016) predicted the possible c-ring rotational mechanism as follows: (1) only the *i*th c-subunit is deprotonated [denoted as DEP(c_i_)]; (2) the protonated *j*th c-subunit releases protons to the matrix region; (3) c-ring rotates; (4) the *i*th c-subunit receives the proton from the IMS region; and (5) c-ring rotates and completes the 36° step rotation (Leone and Faraldo-Gómez, 2016). This mechanism can be represented as follows:


$$\begin{array}{l} \text{DEP}(c_{i})\overset{\text{deprot}}{\to }\text{DEP}(c_{i},\;c_{j})\overset{\text{rot}}{\to }\text{DEP}(c_{i},\;c_{j})\overset{\text{prot}}{\to }\text{DEP}(c_{j})\\ \overset{\text{rot}}{\to }\text{DEP}(c_{j}) \end{array}$$

In contrast, our MC/MD simulations (Kubo et al., 2020) and recent all-atom MD simulations (Marciniak et al., 2022) based on recent high-resolution structures suggested a new pathway, in which two or three c-subunits are simultaneously deprotonated under rotation, as shown in Figure 1B (right). This pathway can be described as follows:


$$\begin{array}{l} \text{DEP}(c_{i},\;c_{j})\overset{\text{deprot}}{\to }\text{DEP}(c_{i},\;c_{j},\;c_{a})\overset{\text{rot}}{\to }\text{DEP}(c_{i},\;c_{j},\;c_{a})\\ \overset{\text{prot}}{\to }\text{DEP}(c_{j},\;c_{a})\overset{\text{rot}}{\to }\text{DEP}(c_{j},\;c_{a}) \end{array}$$

The results of our MC/MD simulations are described in greater detail below. Discrepancies between the two pathways may be due to the different species (the former was for F_O_ in *Polytomella* sp. and the latter for F_O_ in yeast). If two half-channels are close to each other, there is no space for an additional deprotonated c-subunit. Alternatively, these differences may arise from the resolution of the original cryo-EM maps (~6.2 vs. 3.6 Å).

The hybrid MC/MD approach has many benefits for understanding mechanisms of molecular motors, in general. It can track conformational dynamics together with some elementary reactions, such as proton movements in the F_O_ motor. We have succeeded in theoretically reproducing the rotational motion of the F_O_ motor corresponding to the proton motion by combining MD and MC. Remarkably, the model that used the hybrid MC/MD approach rotated with a force equivalent to the torque confirmed in the experiment. However, it should be noted that these methods were based on many assumptions and have many limitations. Coarse-grained MD does not take into account the side chains orientation, and the simulated time correspondence with the real time is not perfect. All-atom MD can solve these problems, but it is hard to handle the entire motion of a huge system such as a F_O_F_1_ motor at once because of the computational cost. The MC methods were able to treat the proton movement only empirically; nothing that is not designed happens. In principle, it is possible to handle the proton transfer by the hybrid quantum mechanics/molecular mechanics simulation, but the computational cost is way beyond the current reach. The hybrid MC/MD simulation we performed is a sort of compromise. Along this line, the constant-pH MD simulation is a closely related approach, in which protonation states in relevant amino acids are updated *via* MC processes. However, the commonly used constant-pH MD simulation allows appearance and disappearance of protons at any protonatable amino acids instead of considering the proton transfer from one site to the other. Thus, this cannot directly be used in the proton-transport study. This can be modified in a similar manner to the introduced hybrid MC/MD approach. Despite much room of improvement, the hybrid MC/MD approach has the advantage of being able to track proton and conformational motions with a high spatiotemporal resolution than experiments.

## Proton Transfer Pathway Based on Atomic-Resolution Structure Model

Several high-resolution structural models have been obtained using cryo-EM, allowing unambiguous discussions on proton transfer pathways. Klusch et al. (2017) identified water-accessible channels in their mitochondrial ATP synthase structure model used the program Hollow (Ho and Gruswitz, 2008). Based on the water-accessible region and molecular model constructed from a map at 3.7 Å resolution, plausible proton transfer pathways across the membrane could be investigated. First, the pathway from the IMS to the c-ring involves the following two steps. (1) From the IMS aqueous environment, protons enter the a-subunit through the aqueous space around aE172, aH248, aH252, and aE288 (yellow oval in Figure 1C). (2) Then, protons pass through the space between aN243 (H5) and aE288 (H6), reaching the side chain of cE111 (chain-i in Figure 1B), which is protonated (the proton-carrier residue of the c-ring). The water-accessible region ends around aR239, which is a strictly conserved arginine residue of the a-subunit located between the two half-channels. Based on this pathway, aR239 plays a role in preventing direct proton jump between the two half-channels, which corresponds to proton leakage. Next, the pathway from the c-ring to the matrix-side aqueous space likely involves the following steps. (1) The proton-carrying cE111 (chain-a in Figure 1B) moves from the lipid-facing environment to the region that contacts the a-subunit (particularly H5) *via* c-ring rotation. (2) Then, cE111 releases the protons to exit into the matrix space. Because the side chain of aE225(H5) faces the c-ring and is expected to function as the end of the half-channel that reaches the matrix-side aqueous space, the proton passes from aE225 to the inside of the a-subunit and is released into the matrix space (yellow oval in Figure 1C). Notably, the orientation of the funnel-like space with H4–loop–H5 of the a-subunit with respect to the c-ring should be unaltered. Especially, it may be so essential for ATP synthase activity that H5 is positioned along the c-ring as discussed in (Klusch et al., 2017). For because residues aW189(H4), aT193(H4), and aY229(H5) are thought to contribute to maintaining the angle of the funnel-like tip, and mutations of these residues (or those present at the same position in other species) resulted in severe mitochondrial diseases (De Meirleir et al., 1995; Castagna et al., 2007; Cortés-Hernández et al., 2007). The mutations might cause wrong orientation of the H5 curvature along the c-ring, it would separate aE225 (matrix channel) from cE111 (proton carrier of the c-ring), and the distance between the c-ring and the channel will make it difficult for protons to transport. By the way, the subtlety of this pathway warrants discussion. Roh et al. (2020) identified a tightly bound lipid molecule in the cavity between the c-ring and a-subunit in the cytosol of the V_O_ motor (corresponding to the matrix side in the case of mitochondrial F_O_). This tightly bound lipid molecule prevents the direct access of water molecules from the cytoplasm to the c-ring. It should be noted that V_O_ motor conformation has some notable difference from those of the F_O_ motor. For example, one of the c-subunits in the V_O_ is different from the others. Thus, it is not clear how well the mechanisms of Vo can be generalized to F_O_. Therefore, it is unlikely that protons are directly released by the c-ring proton-carrying residues into the matrix-side aqueous space, as anticipated in Fo. Instead, the proton in the c-ring residue would be first transferred to the a-subunit proton-carrying residue, followed by release on the matrix side.

**Figure 2:**
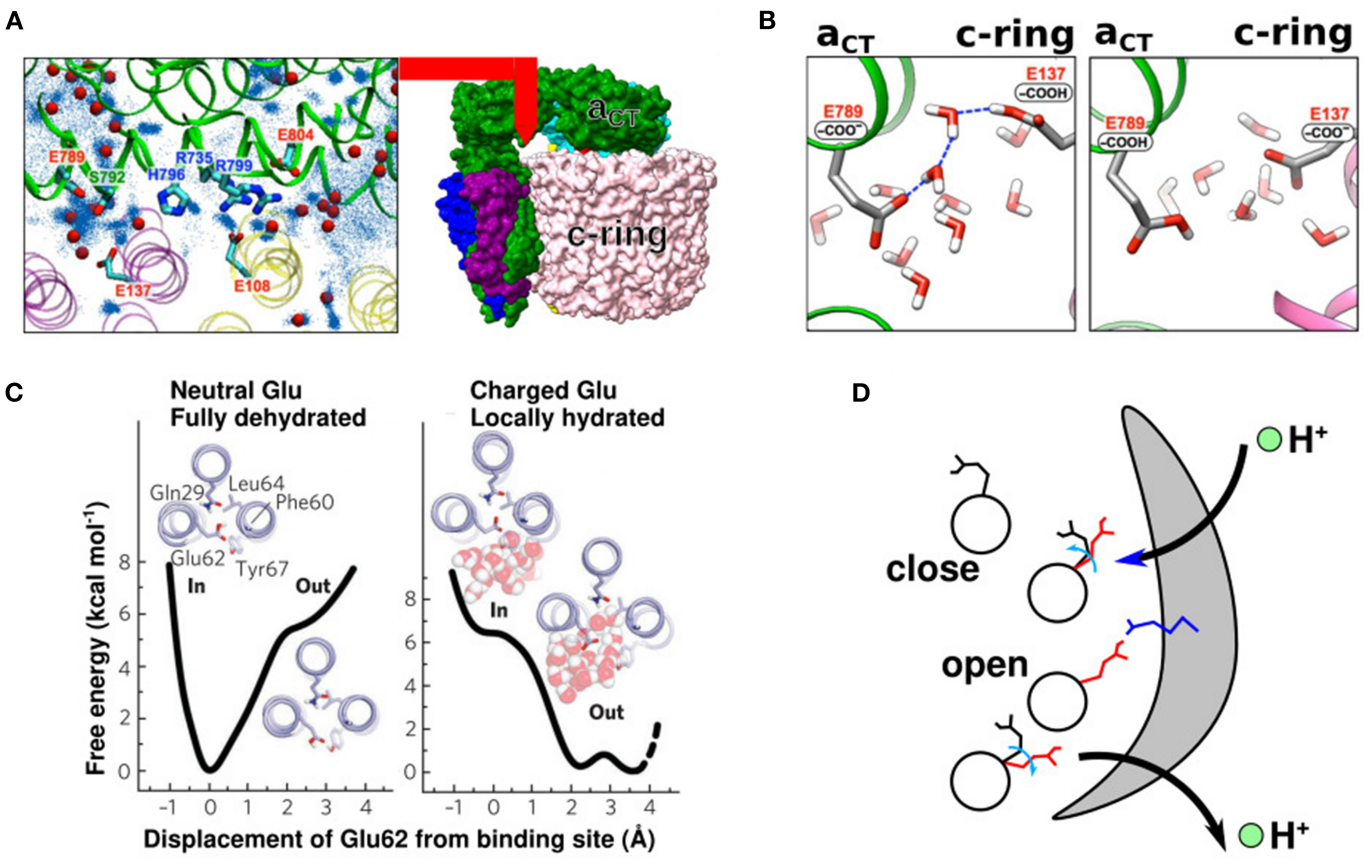
Side-chain conformation coupled with the protonation states. **(A)** A 2.7 map of the V_O_ motor a-subunit (green) and c-ring (pink and yellow) (right panel). Water molecules near the interface between the α-subunit and c-ring (left panel). Red beads are defined by cryo-EM map and Coot function. Blue dots are defined by all-atom MD simulation (Roh et al., 2020) **(B)** Representative snapshot of all-atom MD simulation with protonated (left) and deprotonated (right) cE137. The left panel shows a water-wire (Roh et al., 2020) **(C)** Free energy surfaces of the closed Pogoryelov et al. (2010) and open conformations of cE62 in *S. platensis*. **(D)** Proton movement with changes in glutamate conformation. Black and red side chains indicate the closed and open glutamate conformation, respectively. Blue side chain indicates the highly conserved arginine of the a-subunit.

If atomic-resolution electron maps are available, water molecules can be directly traced to the channels. Roh et al. (2020) marked water molecules on a 2.7 Å map using the “Find Waters” routine available in Coot (Emsley et al., 2010) (blue dots in Figure 2A). The authors also performed all-atom MD simulations from a randomly arranged water molecule configuration and mapped the frequency of the water molecule positions (red spheres in Figure 2A). Thus, Coot mapping, in conjugation with all-atom MD simulations, is an effective method to evaluate where water molecules are released inside the water-accessible channel. The authors also found that water molecules reside between the half-channels and c-subunit, possibly enabling proton relay between the a- and c-subunits. Interestingly, the placement of relaying water molecules strongly depended on the protonation state of the proton-relaying residues; thus, the authors examined two different setups for the protonation states of glutamate. In the first setup, cE137 (the proton-carrying residue of the yeast V_O_ motor c-ring) was protonated and aE789 (the representative glutamate on the lumen side half-channel of the yeast V_O_ motor) was deprotonated (left panel in Figure 2B). In the second setup, the protonation states of the two glutamates were swapped (right panel in Figure 2B). A “water wire”—a single-file arrangement of water molecules—was stably formed between the protonated cE137 and the deprotonated aE789 (left panel in Figure 2B), but not in the other setup. Unidirectional transport of protons may occur through this wire.

Pogoryelov et al. (2010) illustrated the structure of proton-coupled c-ring in *S. platensis*, which gives structural insights on the proton-transfer pathway. The side chains of the c-ring proton-carrier residues assume variable conformations depending on the protonation state and surrounding environment. Moreover, all-atom MD simulations confirmed that the orientation of the side chains of c-ring acidic proton-carrying residues changes depending on whether the residue faces the lipid membrane or a-subunit. When it faces the lipid membrane, the protonated side chain of the c-ring proton-carrier residue tends to face the inner side of the c-ring (proton-locked conformation) (left panel in Figure 2C). Meanwhile, when it faces the a-subunit, the deprotonated side chains face outward (deprotonated-extended conformation), facilitating proton transfer from the c-ring to the half-channels of the a-subunit (right panel in Figure 2C). These two conformations have also been reported in yeast V_O_ motors (Roh et al., 2020). Overall, the proton relay can be summarized as follows (Figure 2D). (1) The c-subunit glutamate in the membrane-facing region is protonated and exists in a closed state. (2) When it faces the a-subunit, it releases protons, undergoing deprotonation-coupled conformational change of the side chain to the open state. (3) When the deprotonated glutamate comes close to the other half-channel *via* c-ring rotation, it receives a proton, followed by a conformational change to the closed state. (4) Upon protonation, glutamate in the closed state enters the lipid-facing region.

## Free Energy Profiles of Protons and pKa Values

ATP synthesis by the action of F_O_F_1_ ATP synthase is driven by the proton motive force, which is the sum of contributions from the proton concentration gradient and membrane electrical potential as pointed out by Mitchell (1961). For the former, the pH values on the IMS and matrix sides are ~7.0 and 8.0, respectively, corresponding to a free energy difference of 2.3 *k*_*B*_*T*. Typically, the mitochondrial membrane electrical potential is 150 mV, corresponding to 5.4 *k*_*B*_*T*. Notably, Soga et al. (2012) found that the sum of the two factors, namely the contribution from the pH difference across the membrane and membrane electrical potential, rather than their individual contribution is relevant to ATP synthesis kinetics. This suggests that not only the thermodynamics, but also kinetics are largely controlled by the sum of the two contributions. Notably, Soga et al. (2012) used a bacterial ATP synthase that was engineered with a deletion of an endogenous inhibitory domain (epsilon subunit's C-terminal domain), so they eliminated another factor that might control kinetics *in vivo*.

To understand the free energy profile of proton transfer through the transport pathway, in addition to the overall driving force, another key factor is the intrinsic affinity of protons toward every proton carrier residue; this is quantified in terms of the pKa value, which is proportional to the protonation free energy of the corresponding chemical group and calculated as *V*_*pKa*_ = −(*ln* 10) *k*_*B*_*T pKa*.

Based on high-resolution structures, Srivastava et al. (2018) discussed the pKa values of glutamate residues of two half-channels in yeast ATP synthase (aE223 for the IMS channel and aE162 for the matrix channel) and the c-ring glutamate responsible for proton transport (cE59). If the pKa of glutamate is 5.0, the standard value in an aqueous solution as they mentioned in the paper, the difference in pH on the matrix side would be 3.0. Note, glutamate pKa value in water is, typically, treated around 4 (Tollinger et al., 2002). In this case, proton transfer from aE162 to the matrix side during the ATP synthesis reaction would be easy, although reverse proton transfer during ATP hydrolysis would be difficult. Thus, the authors inferred that the pKa values of glutamates and c-ring proton-carrier residues of the two half-channels should be ~7.0. The pKa value of carboxylates shifted to ~7.0 through glutamate–glutamate and histidine–glutamate interactions (Koeppe and Stroud, 1976; Root and Mackinnon, 1994; Morrill and MacKinnon, 1999; Harris and Turner, 2002). In addition, although the distance between the side chains of cE59 and aE223/aE162 was not small in their structure, they confirmed that it could be brought to ~4 Å through specific c-ring rotation. Therefore, the authors speculated that c-ring rotation brings cE59 and aE223/aE162 closer, shifting the pKa value to near 7.0. In fact, as shown in Figure 2D, when the c-ring is close to the a-subunit, the orientation of its proton-carrier side chains changes in the direction in which the reaction is more likely to occur; therefore, the shift in pKa values is expected to occur at the same time. Most recently, Marciniak et al. (2022) calculated the shift in pKa values of glutamates using all-atom MD simulations and reported that pKa values of the matrix and IMS sites faced c-subunits' glutamates were ~2 and 8, respectively. Of note, however, accurate *de novo* estimation of the pKa value is very challenging owing to its sensitivity to the local environments and approximations used.

## Free Energy Profiles Along Rotary Angles for Given Protonation States

**Figure 3:**
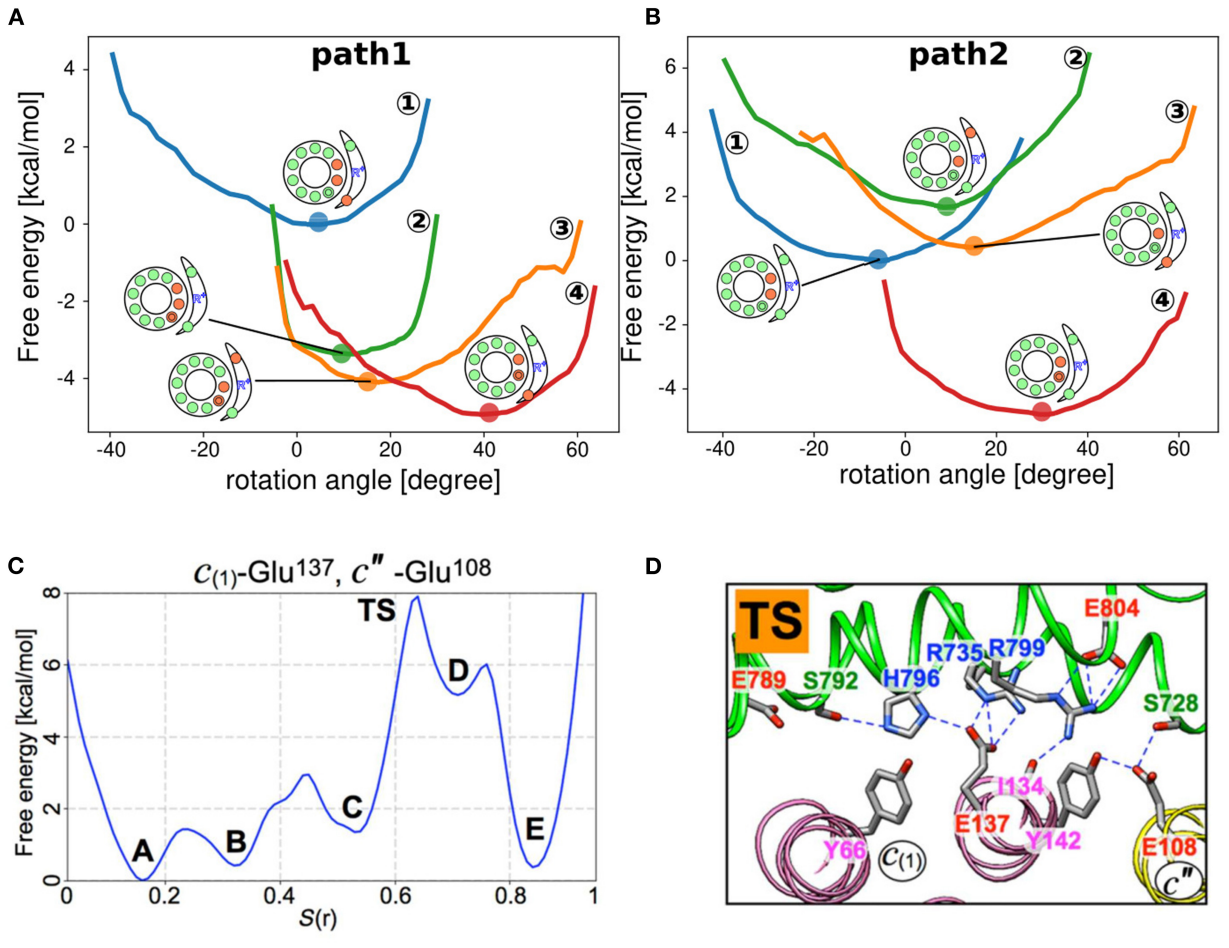
Free energy surface based on the rotational angle. **(A,B)** Free energy surfaces of individual protonation states obtained from coarse-grained MD simulations (Kubo et al., 2020). “Path 1” and “Path 2” represent the deprotonation-first and the protonation-first pathways, respectively. **(C)** Free energy surface of the c-ring rotation in yeast V_O_ motor. Five local minima (A–E) and transition states (TS) (Roh et al., 2020). **(D)** Representative snapshot of the TS state in **(C)**. The a-subunit and c-ring interface is focused, with some polar residue side chains indicated with sticks. Blue dashed lines indicate hydrogen bonds (Roh et al., 2020).

In previous sections, we discussed how the protonation and deprotonation of glutamates in the F_O_ motor are controlled by the environment, such as pKa, pH, and membrane potential, as well as physical interactions, such as whether they faces the a-subunit or lipid molecules. Now, we shall focus on the actual free energy of the F_O_ motor along the rotary angle. Recently, for some representative protonation states obtained from our MC/MD simulations (details are described later), we drew the free energy surfaces of different protonation states (Figures 3A,B) (Kubo et al., 2020). For each fixed protonation state, we performed coarse-grained MD simulations to obtain the free energy surface. Using these trajectories, we employed the Bennett acceptance ratio method (Bennett, 1976) and calculated the free energy differences among different protonation states. Based on the results, we illustrated the preferred proton transfer pathways for both ATP hydrolysis and ATP synthesis. Starting from the state in which the proton-carrying residues of the two c-subunits are deprotonated, we propose two plausible pathways of ATP synthesis: the deprotonation-first pathway (Figure 3A) and the protonation-first pathway (Figure 3B). We speculate that the deprotonation-first pathway occurs more frequently than the protonation-first pathway. Warshel group also obtained free energy surfaces using fixed protonated states and coarse-grained MD; Using Metropolis MC proton transfer approach (Vorobyov et al., 2016) to determine the protonation state of each c-subunit of the chloroplast ATP synthase F_O_ motor and determined the free energy surface for each angle of the c-ring (Bai and Warshel, 2019).

Using all-atom MD simulations, Roh et al. (2020) determined free energy surfaces for c-ring angles based on the yeast V_O_ motor structures they obtained. The authors selected a few representative residues for (de)protonation and rotated the c-ring for each protonation setup by adding a force to the residues. The string method was used to refine the pathway. Finally, 50 intermediate structures along the pathway were determined in a rotation of ~14° and connected by successive all-atom MD simulations. Successive all-atom MD simulations revealed that the free energy surface of c-ring rotation showed five intermediate states (A to E) and a transition state (TS) between states C and D (Figure 3C). At the TS, c^′′^E108 completely lost hydrogen bonds with aR735 and aR799 (Figure 3D). These contacts were maintained until state C. Thus, the loss of these connections is the rate-limiting step in the reaction. In addition, Marciniak et al. (2022) determined free energy surfaces depending on the c-ring rotational angle for the yeast F_O_ motor.

Technically, although coarse-grained MD simulations can easily sample various conformations, they are intrinsically less accurate. Meanwhile, while all-atom MD simulations are more accurate, they are limited in sampling for large molecular machines, such as ATP synthase. In a recent study (Unarta et al., 2021), the merits of the two approaches were combined: a wide range of structures sampled by coarse-grained MD simulations were back-mapped to all-atom models, followed by all-atom MD simulations.

## Proton Transfer-Coupled F_O_ Rotation

Furthermore, we investigated how proton transfer is dynamically coupled to c-ring rotation. The rotation of the F_O_ motor is realized through proton transfer between the two half-channels and the c-ring *via* protonation and deprotonation of their side chains. In particular, the electrostatic interaction between the conserved arginine residue of the a-subunit and the c-subunit acidic proton-carrying residue is thought to be important in driving the rotation of the F_O_ motor during ATP synthesis. However, how they are dynamically coupled remains unclear.

**Figure 4:**
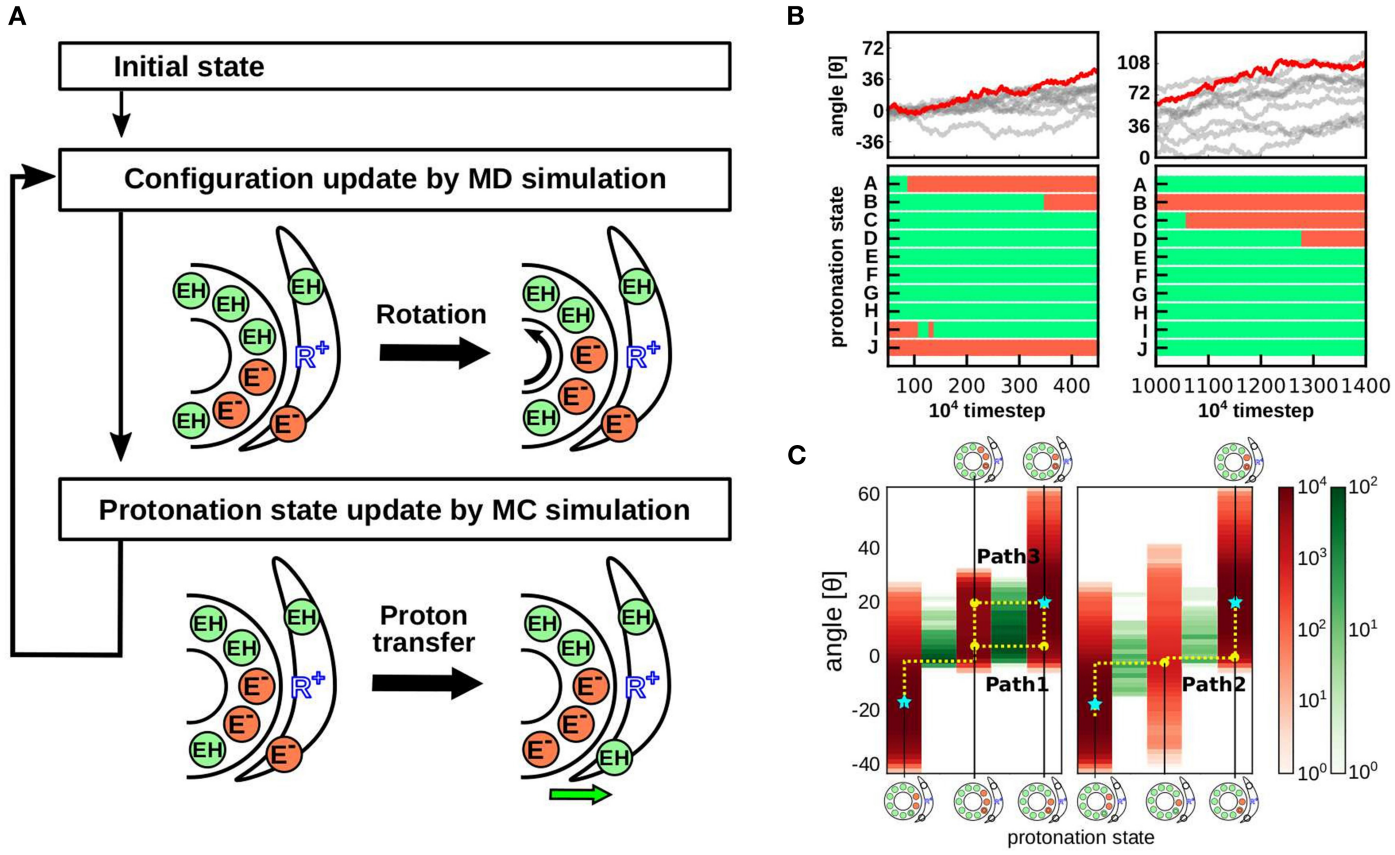
Hybrid MC/MD simulation protocol and results. **(A)** Our hybrid MC/MD simulation protocol. We performed alternative short MC and MD simulations. **(B)** Representative trajectory of the cumulative c-ring rotational angle (top) and protonation state of each c-subunits (bottom). The bottom time courses are obtained from the red trajectory in the top panels. Protonated and deprotonated states are indicated in green and orange, respectively. **(C)** Protonation state distribution depends on the c-ring rotational angle. The rotational angle is indicated in red, and the proton transfer event at each angle is indicated in green (Kubo et al., 2020).

Recently, we performed hybrid Metropolis MC/MD simulations, where proton transfer was mimicked by an MC move and the rotational motion of the F_O_ motor was realized through MD simulations (Figure 4A) (Kubo et al., 2020). We conducted MD and MC steps alternatively; in the MD step, only the protein conformations were updated, while in the MC step, the protonation state for a fixed conformation was updated. In this section, we briefly describe the computational approach.

We used the yeast mitochondrial F_O_ structure (PDB ID: 6CP6) (Srivastava et al., 2018). Our simulation system comprised an a-subunit and 10 c-subunits, forming a c10-ring. The proton carrier residue in the c-subunit was cE59. In the IMS and matrix channels, the residues that directly transferred protons to and from the c-ring were aE223 and aE162, respectively. We defined these 12 glutamates (10 cE59 residues and 2 residues in the half-channels) as proton-carrier sites and dynamically updated their protonation states. Their protonation states are represented by a 12-dimensional vector $\left\{h_{i}^{+}\right\}$, where each element $h_{i}^{+}$ is either zero when protonated or one when deprotonated. For simulation, we assigned -$h_{i}^{+}$ charge to each glutamate.

The total energy function was defined as follows:


$$V_{total}(\text{R},\;{\text{H}}^{+})=V_{non-es}(\text{R})+V_{es}(\text{R},\;{\text{H}}^{+})+V_{pKa}({\text{H}}^{+})$$

where R represents all protein structure coordinates. On the right-hand side, the first term is the protein interaction energy, except for electrostatic interactions, and it is, therefore, independent of the protonation state (H^+^); the second term is the electrostatic interaction, which depends on both the conformation and protonation state; and the last term represents the intrinsic preference for protonation, corresponding to the intrinsic pKa, which is assumed to be independent of the conformation.

During the MD step, protein conformation dynamics were determined as *V*_*non*−*es*_(R) and $V_{es}(\text{R},\;{\text{H}}^{+})$. For *V*_*non*−*es*_(R), we used a well-established structure-based model that maintains both c-ring and a-subunit structures (Li et al., 2014). This model uses a coarse-grained representation of proteins, where each amino acid is simplified as one bead located at the Cα position. $V_{es}(\text{R},\;{\text{H}}^{+})$ includes two terms: the Coulomb interaction between any charged residue term $V_{C}(\text{R},\;{\text{H}}^{+})$, and the membrane-environment term $V_{mem}(\text{R},\;{\text{H}}^{+})$. The latter term approximates the electrostatic penalty to embed a charged residue, deprotonated glutamate in this case, into the lipid membrane environment. The simulations did not explicitly include lipid molecules. Instead, we assumed that the c-ring glutamate faces the lipid membrane not the a-subunit. When we reduced the lipid membrane area in the control simulation, rotation activity was markedly reduced, which suggests the importance of the funnel-like structure of the matrix-side half-channel, corroborating the view put forth by Klusch et al. (2017). Approximation of the lipid membrane area can be refined using all-atom models. For instance, using the CHARMM-GUI membrane builder (Jo et al., 2008; Wu et al., 2014; Lee et al., 2016), lipid molecules can be explicitly placed around the F_O_ motor, the range within which lipids are present can be estimated, and the free energies of protonation/deprotonation can be obtained. In a recent study, Marciniak et al. (2022) reported this direction. Also, Novitskaia et al. (2019) used CHARMM-GUI membrane builder for evaluation of the number of lipid molecules inside c-ring.

The MC step begins with probabilistic selection of the protonation states of two glutamates in the a-subunit, namely aE223 and aE162, for yeast mitochondrial ATP synthase, followed by probabilistic proton transfer between the a-subunit and c-subunit glutamates. Here, an underlying assumption is that water molecules in the half-channels can move protons fast enough to equilibrate the protonation states of aE223 and aE162 for the IMS and matrix-side half-channels, respectively. The protonation energy for each residue can be calculated as follows:


$$\begin{array}{l} \epsilon_{aE223}\equiv \left(ln10\right)k_{B}T\left(pH_{IMS}-pKa_{aE223}\right)-\Delta \text{Ψ}/2\\ \epsilon_{aE162}\equiv \left(ln10\right)k_{B}T\left(pH_{matrix}-pKa_{aE162}\right)+\Delta \text{Ψ}/2 \end{array}$$

The pH values of the IMS and matrix aqueous space were set to 7.0 and 8.0, respectively, and the membrane potential (Ψ) to 150 mV. As all glutamates are located near the center of the membrane bilayer, we assumed the electrical potential at that site to be Ψ/2. Accordingly, the local equilibrium probabilities of protonation at each site *P*_*i*_ can be calculated as follows:


$$\begin{array}{l} P_{i}=\frac{\exp\left(-\epsilon_{i}/k_{B}T\right)}{1 + exp\left(-\epsilon_{i}/k_{B}T\right)} \end{array}$$

where *i* = aE223 or aE162; each MC phase begins with the probabilistic selection of the protonation states of aE223 and aE162 with these values.

Next, we realized probabilistic proton transfer between the c-subunit and a-subunit glutamates. For proton transfer between pairs, the following conditions must be satisfied: (1) only one glutamate molecule in each pair is protonated; (2) the two glutamates are close to each other; (3) they are located at an appropriate angle; and (4) they are not located across aR176, which is a highly conserved arginine in the a-subunit that separates the two half channels. The kinetic weight factor for the three last criteria can be given as follows:


$$\begin{array}{l} w_{j\leftarrow i}\left(r,\;\theta \right)=f\left(r\right)g\left(\theta \right)h_{aR176} \end{array}$$

where *f*(*r*) is the decreasing function of the distance *r* between two glutamates; *g*(θ) is the function of angle θ between the two vectors that emulate the side chain conformation of the donor and acceptor; *g*(θ) assumes a Gaussian form centered around the corresponding angle in the cryo-EM structure; and *h*_*aR*176_ represents the role of aR176 in preventing proton leakage between two half channels. We assumed that *h*_*aR*176_ was 0 when the proton donor and acceptor were located across aR176; otherwise, *h*_*aR*176_ = 1.

Using the kinetic weight factor *w*_*j*←*i*_, the probability of proton transfer from the *i*^th^ site to the *j*^th^ site can be given as follows:


$$\begin{array}{l} \begin{array}{l} p\left({\text{H}}^{+\left(before\right)}\to {\text{H}}^{+\left(after\right)}\right)\\ \;=(1-e^{-k\tau w_{j\leftarrow i}})Min(1,\;\exp(-\Delta E/k_{B}T)) \end{array} \end{array}$$

where *E* is the energy change upon proton transfer; *k* is the overall rate constant; and τ is the time step between MC trials. We set τ = 10^5^ MD steps. As the value of the overall rate constant of proton transfer *k* is unknown, we simply set *kτ* = 1. H^+^(*before*) and H^+^(*after*) represent the protonation states before and after the proton transfer trial, respectively. Accordingly, energy change upon proton transfer can be given as follows:


$$\begin{array}{l} \Delta E=V_{total}\left(\text{R},\;{\text{H}}^{+\left(after\right)}\right)-V_{total}\left(\text{R},\;{\text{H}}^{+\left(before\right)}\right) \end{array}$$

where the second and third terms in *V*_*total*_ are relevant to proton transfer, and the last term $V_{pKa}\left({\text{H}}^{+}\right)$ represents the intrinsic propensity for protonation at each site (i.e., 2 half-channels and 10 c-subunits). In a previous study (Kubo et al., 2020), we set *pKa*_*aE*223_ = 6.0, *pKa*_*aE*162_ = 9.0, and *pKa*_*cE*59_ = 8.0, based on discussion by Srivastava et al. (2018). The reliability of these values was also examined using the pKa calculation tool PROPKA (Li et al., 2005). Note, the all parameter used in our previous study are listed in Table 1.

**Table 1:** All parameter list tested in Kubo et al. (2020).

	**Kubo et al. (2020)**	**Test in Kubo et al. (2020)**
pH (IMS; aE223)	7.0	
pH (matrix; aE162)	8.0	7.0, 9.0
pKa (aE223)	6.0	
pKa (cE59)	8.0	
pKa (aE162)	9.0	8.0, 7.0
Membrane potential	150 mV (for cE59)	0, 300

## Two or Three c-Subunits are Deprotonated During a Single Rotation Step

Earlier theoretical models assume that after a proton is released from the c-subunit into the matrix, a single-step rotation of the c-ring is followed by the receipt of a proton from the IMS site to the same or the nearest neighbor c-subunit (Elston et al., 1998) (left panel in Figure 1B). In other words, during the cycle, the number of deprotonated proton-carrier residues in the c-subunit is between zero and two. Subsequently, high-resolution cryo-EM structures revealed the accurate position of the highly tilted α-subunit, suggesting that the deprotonated c-subunit is be immediately protonated in the next step (right panel in Figure 1B). In fact, our hybrid MC/MD simulations indicated that proton-carrier residues in the two c-subunits are simultaneously deprotonated in the ground state (Figure 4B). In addition, a recent study using all-atom MD simulation supports the presence of doubly deprotonated c-subunits (Marciniak et al., 2022).

Starting from the doubly deprotonated c-ring configuration, which occurs next: further deprotonation to the triple-deprotonated state (deprotonation-first pathway) or protonation to the single-protonated state (protonation-first pathway)? How is this coupled to c-ring rotation? Based on free energy profiles obtained from our simulations along the two scenarios (Figures 3A,B), the deprotonation-first pathway has a lower free energy cost, whereas the protonation-first pathway requires high free energy to achieve the single-protonated state. Interestingly, the deprotonation-first pathway was dominant in both ATP synthesis and hydrolysis. However, the proposed pathway does not fully consider the dynamical aspects of c-ring rotation coupled to proton transfer. Thus, through further dynamic hybrid MC/MD simulations, we obtained the most prominent pathway in the synthetic direction (Figure 4C) (Kubo et al., 2020), as follows:


$$\begin{array}{l} \text{DEP}\left({\text{c1}}_{\text{1}},\;{\text{c2}}_{\text{2}}\right)\;\theta \;\sim \;-{\text{18}}^{{}^{\circ}}\overset{\text{rot}}{\to }\text{DEP}\left({\text{c}}_{\text{1}},\;{\text{c}}_{\text{2}}^{{}^{\circ}}\right)\;\theta \;\sim \;-{\text{2}}^{{}^{\circ}}\overset{\text{deprot}}{\to }\\ \text{DEP}\left({\text{c}}_{\text{1}},\;{\text{c}}_{\text{2}},\;{\text{c}}_{\text{3}}\right)\;\theta \;\sim \;-{\text{2}}^{{}^{\circ}}\overset{\text{rot}}{\to }\text{DEP}\left({\text{c}}_{\text{1}},\;{\text{c}}_{\text{2}},\;{\text{c}}_{\text{3}}\right)\;\theta \;\sim \;{\text{3}}^{{}^{\circ}}\overset{\text{prot}}{\to }\\ \text{DEP}\left({\text{c}}_{\text{2}},\;{\text{c}}_{\text{3}}\right)\;\theta \;\sim \;{\text{-3}}^{{}^{\circ}}\overset{\text{rot}}{\to }\text{DEP}\left({\text{c}}_{\text{2}},\;{\text{c}}_{\text{3}}\right)\;\theta \;\sim \;{\text{18}}^{{}^{\circ}} \end{array}$$

In this pathway, mean c-ring rotational position in the double-deprotonated state *DEP*(*c*_1_, *c*_2_) is at −18, with marked fluctuations (here, the reference of the angle is arbitrary; therefore, the change in angle rather than its absolute value is important). When the value fluctuates to −2, the third c-subunit *c*_3_ releases a proton, resulting in the triple-deprotonated state *DEP*(*c*_1_, *c*_2_, *c*_3_), with relatively limited fluctuations. After a while, the c-subunit *c*_1_receives a proton, followed by c-ring rotation to an angle of 18.

Currently, this pathway has not been directly confirmed by other studies. Although very challenging, direct observations of rotational motions together with proton transfer are warranted. However, a recent study demonstrating the coupling among neighboring c-subunits supports the involvement of a multiply deprotonated state, as described below (Mitome et al., 2022).

## F_O_-asp Mutants Exhibited a Characteristic Decrease in Activity

**Figure 5:**
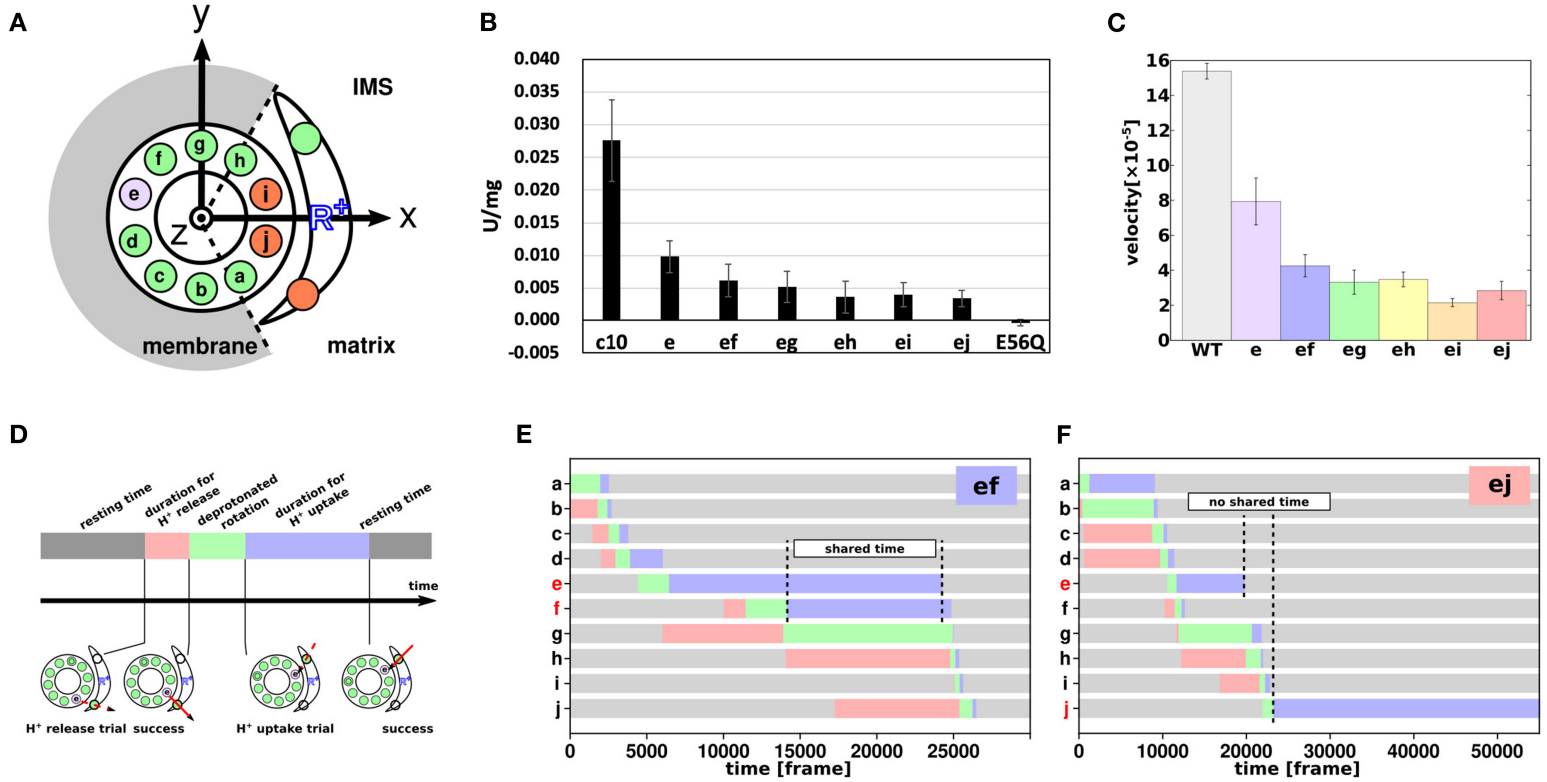
Coupling among c-subunits for F_O_ rotation. **(A)** Schematic cartoon of the a-subunit and c-ring of the F_O_ motor. The 10 c-subunits are labeled a–j. Circles represent 10 glutamates of the c-ring and 2 glutamates of the IMS and matrix channels. Green and orange represent protonated and deprotonated states, respectively. Purple represent cE56D mutant c-subunit. **(B)** ATP synthesis driven by NADH oxidation. c10 is the wild-type (WT); e to ej are the results of cE56D mutants, and E56Q are the results of c10(E56Q)- F_O_F_1_. Bars represent standard error. **(C)** Average rotational velocities for WT and mutants in coarse-grained MC/MD simulations. Bars represent standard error. **(D)** Cartoon showing the four phases of proton transfer, namely the resting time (gray), proton release duration (red), deprotonated rotation (green), and proton uptake duration (blue). **(E,F)** Representative time course of durations for the double mutant “ef” in **(E)** and “ej” in **(F)** (Mitome et al., 2022).

Proton transfer-coupled molecular simulations suggested that proton-carrier residues in two or three c-subunits are simultaneously deprotonated while the F_O_ motor rotates. Recently, we examined the coupling of multiple c-subunits in F_O_ through a collaborative study of biochemical assays and molecular simulations of mutant F_O_ motors (Mitome et al., 2022). First, Mitome et al. (2004) built a genetically fused single-chain c-ring of *Bacillus* PS3 ATP synthase. In the single-chain c-ring, 10 c-subunits were labeled in the order “a” through “j” (Figure 5A). We then introduced a mutation of the proton-carrier cE56 to aspartic acid in a specific c-subunit of the single-chain c-ring. Due to its shorter side chain and lower pKa value, the cE56D mutation in the c-subunit “e” slowed down yet did not abolish ATP synthesis compared with the wildtype (c10 and “e” in Figure 5B). Furthermore, double mutants carrying cE56D mutations in two c-subunits exhibited further reduced ATP synthesis activity compared with the single mutants. Interestingly, the ATP synthesis activity in double mutants depended on the distance between the two mutated c-subunits. Specifically, among the five double mutants examined, the ones carrying mutations in the neighboring c-subunits (“ef”) exhibited the highest ATP synthesis activity. The activity tended to decrease with increase in distance between the two mutated subunits. Motivated by this finding, we performed molecular simulations on the corresponding mutant of mitochondrial F_O_F_1_ ATP synthase that we had used earlier and found that the F_O_ rotation rate decreased as the distance between two mutation sites increased, which is consistent with the results of biochemical assays (Figure 5C). Although this experiment alone did not offer any direct mechanistic understanding, the corresponding simulations provided some clues. Briefly, four phases were defined for each c-subunit: resting time, proton release duration, deprotonated rotation, and proton uptake duration (Figure 5D). The time series of the 10 c-subunit protonation states was plotted based on the four phases defined. Figures 5E,F show the four phases of “ef”, in which the neighboring “e” and “f” are mutated, and “ej”, in which the farthest subunits “e” and “j” are mutated. The time for the most prolonged duration for proton uptake was markedly shared in “ef” (Figure 5E), but not in “ej” (Figure 5F). Thus, the farther apart the mutated c-subunits are, the less latency they share for protonation and the lower is their activity. Thus, the results of biochemical assays are consistent with the view that the proton-carrier residues in multiple c-subunits are simultaneously deprotonated, indicating coupling among the c-subunits.

## Mutation of the Conserved Arginine of the a-Subunit

As previously described, mutations of the c-subunit proton-carrier residues as well as of the a-subunit affect rotational activity of the F_O_ motor. The effect of mutation of the highly conserved aArg (aR176 in mitochondrial ATP synthase) was significant. From structural information, aArg is important for separating the two water-accessible regions beyond the two half-channels. In addition, Mitome et al. (2010) found that aArg mutation decreased ATPase activity and induced proton leakage. Thus, we tested leak events through simulations mimicking the aR176A mutation. We modeled the effect of the arginine mutation by replacing the factor *h*_*aR*176_ with unity. Consequently, both ATP synthesis and hydrolysis were markedly suppressed. In particular, in the ATP hydrolysis pathway, protons leaked in the opposite direction. Proton leakage occurred both in ATP synthesis and hydrolysis during the waiting time for the c-subunit to receive protons (Kubo et al., 2020).

Using all-atom MD simulations, Marciniak et al. (2022) found that the aR176A mutation connects the two half channels such that water molecules can directly pass through, regardless of the c-ring rotation. The authors also noted similar results in the aR176K mutant, which may be due to the higher flexibility of the lysine side chain than that of the arginine side chain.

## Discussion on How to Solve the Symmetry Mismatch

As high-resolution cryo-EM structure models of the F_O_ motor, together with functional and theoretical information, offered a refined view of proton transfer-coupled F_O_ rotation, the next aspect to be refined was how F_O_ rotation is coupled to ATP synthesis. The fundamental mechanism of coupling has long been known; briefly, the F_O_ motor rotates the central rotor, which is shared with the F_1_ motor. The rotation of the central rotor in F_1_, the γ-subunit, induces a series of conformational changes in the F_1_ stator, α_3_β_3_, which drives ATP synthesis at the interface of the α-β-subunits. An obvious question that remains to be addressed is the symmetry mismatch. The F_1_ motor exhibits 3-fold pseudo-symmetry in all species, whereas the F_O_ motor exhibits diverse pseudo-symmetries, ranging from 7- to 13-fold. First, the units of rotational steps in F_O_ and F_1_ are not consistent. Furthermore, the symmetries of F_O_ and F_1_ are coprime in many species, rendering the three unit steps of F_1_ rotation unequal with respect to F_O_ rotation. How does ATP synthase solve this symmetry mismatch issue, realizing high efficiency of energy transduction? Some earlier studies have suggested various possibilities: the elastic torsion of the central rotor may contribute (Cherepanov et al., 1999; Pänke and Rumberg, 1999; Okazaki and Hummer, 2015); the torsion of the peripheral stalk, b-subunit, may be of central importance (Zhou et al., 2015; Guo et al., 2019); or the δ-subunit may absorb the symmetry mismatch (Murphy et al., 2019). Despite various notions, however, no consensus has been reached as yet.

Recently, we sought to elucidate the means of resolving this symmetry through coarse-grained MD simulations for the holo-complex of *Bacillus PS3* F_O_F_1_ ATP synthase (Kubo et al., 2021). In this study, instead of simulating proton transports in F_O_, we mechanically rotated the c-ring with 10 c-subunits at 36°-steps, examining changes in the other parts of the holo-complex structures. Overall, the mismatches between angular positions favored by the F_1_ and F_O_ motors were absorbed by combination of a number of elements, depending on the degree of mismatch. Distortion and twist of the central rotor, γ-subunit, can absorb some mismatches. Distortion of the peripheral stalk, b-subunit, results in the rotation of the F_1_ stator, α_3_β_3_, relative to the F_O_ stator, a-subunit. Furthermore, a comparative study of cryo-EM structure models among three species confirmed the results of MD simulations and elucidated significant deviation of the c-ring rotation angle from the one anticipated based on the symmetry (a multiple of 36°). Notably, in our simulations of the holo-complex, the F_O_ c-ring was forced to rotate at steps of 36° without room for fluctuation; thus, the deviation of the c-ring rotation from its symmetric position could not be observed using this design. However, our previous simulation of proton transfer-coupled F_O_ rotation revealed marked fluctuations in c-ring rotation (Figures 3A,C), which is consistent with the observation of c-ring rotation angle deviation from the ideal value. In the future, hybrid MC/MD simulations for proton transfer-coupled c-ring rotation should be performed on the holo-complex.

## Atomic Details of the Elementary Process of Proton Transfer Coupled to Side Chain Reorientation of Key Residues

As described above, the side chain of glutamates, the proton-carrier residues of the c-ring, assumes two distinct conformations depending on the surrounding environment: open and closed. Changes in conformation induces a pKa shift, and their conformation is important for bidirectional rotation. Thanks to remarkable advances in cryo-EM technology, atomic details of both conformations have already been revealed. In addition, free energy surfaces that depend on both the conformation and surrounding environment have already been estimated. In our MC/MD simulations using a coarse-grained model, we did not treat the side chain explicitly but set the angular relationship to mimic the effect of side chain reorientation.

In the future, more detailed proton transfer pathways and rotational mechanisms of F_O_ motors can be revealed through computational simulations more explicitly taking the orientation of side chains into account. Furthermore, it would be interesting to simulate the actual movement of protons; QM/MM simulations have been employed to explicitly mimic the actual motion of protons during photosynthesis. However, explicit investigations of proton transfer dynamics depending on the rotational motion and orientation of key glutamate side chains is expected to be challenging.

## Author Contributions

SK and ST conceived the project. SK made figures. Both authors were involved in the manuscript writing process. Both authors contributed to the article and approved the submitted version.

## Funding

The study was supported partly by JSPS KAKENHI Grants JP21J00021 (SK), 20H05934 (ST) and 21H02441 (ST), and partly by JPMXP1020200101 as Program for Promoting Researches on the Supercomputer Fugaku (ST).

## Conflict of Interest

The authors declare that the research was conducted in the absence of any commercial or financial relationships that could be construed as a potential conflict of interest.

## Publisher's Note

All claims expressed in this article are solely those of the authors and do not necessarily represent those of their affiliated organizations, or those of the publisher, the editors and the reviewers. Any product that may be evaluated in this article, or claim that may be made by its manufacturer, is not guaranteed or endorsed by the publisher.
